# Lack of Association between *ESR1* and *CYP1A1* Gene Polymorphisms and Susceptibility to Uterine Leiomyoma in Female Patients of Iranian Descent 

**Published:** 2014-05-25

**Authors:** Fatemeh Taghizade Mortezaee, Mohammad Amin Tabatabaiefar, Morteza Hashemzadeh Chaleshtori, Sepideh Miraj

**Affiliations:** 1 Cellular and Molecular Research Center, Shahrekord University of Medical Sciences, Shahrekord, Iran; 2 Department of Genetics and Molecular Biology, School of Medicine, Isfahan University of Medical Sciences, Isfahan, Iran; 3 Deparmment of Obstetrics and Gynecology, Hajar Hospital, Shahrekord University of Medical Sciences, Shahrekord, Iran

**Keywords:** *ESR1*, *CYP1A1*, Uterine Leiomyoma, Polymorphism

## Abstract

Uterine leiomyoma (UL) is the most common benign smooth muscle cell tumor with as yet unknown etiology and pathogenesis. This study was carried out to investigate the association of *ESR1*-351 A>G, *ESR1* -397 T>C and *CYP1A1* (Ile462Val) polymorphisms with UL in female patients of Iranian origin. In this case-control study, 276 patients with UL and 156 healthy women were recruited. The genetic polymorphisms *ESR1*-351 A>G, *ESR1*-397 T>C and *CYP1A1* (Ile462Val) were genotyped by polymerase chain reaction- restriction fragment length polymorphism (PCR-RFLP). No significant difference were found in frequencies of both genotypes and alleles of *ESR1*-351 A>G, *ESR1*-397 T>C and *CYP1A1* (Ile462Val) polymorphisms between the two groups (p>0.05). Our findings indicated that these *ESR1* and *CYP1A1* polymorphisms were not associated with the development of UL in the cases reported here.

Uterine leiomyoma (UL) or fibroid is the most common pelvic benign tumor with a prevalence of 25% among women in the reproductive age and 50% in autopsy reports (1). Pathological investigations have revealed its existence in 73 and 84% of women during premenopause and menopause stages respectively (2). These tumors are the most frequent cause of hysterectomy and uterine surgery. They lead to a variety of symptoms including menstural abnormalities, pelvic pain, infertility and spontaneous abortion (3). Despite the high prevalence of the tumors, exact mechanisms of their initiation and growth remain unclear. Evidence indicates that the growth and the development of UL are estrogen-dependent (4, 5). It has been shown that there is some difference between leyomyoma and normal myometrium in terms of estrogen regulation of gene expression (6), and that leiomyoma tissue is more sensitive to estrogen compared with myometrium. Binding of estrogen to estrogen receptors generate signals in the target cells which prompts cellular proliferation and in the presence of *ESR1* has an anti-apoptotic effect (7). It could also act as a procarcinogen through metabolic processes and induce genotoxicity (8). Several polymorphisms in *ESR1*, located on chromosome 6 (6q24-26), have been reported to be related to gene expression and protein function. There are two well-known polymorphisms in *ESR1*, *ESR1* (XbaI) and *ESR1* (PvuII) both in intron 1 (8, 9). The first step of estrogen metabolism is hydroxylation which is catalyzed by cytochrome P450 1A1 (*CYP1A1*), a member of cytochrome P450 enzymes (10). *CYP1A1* also catalyzes 2-hydroxy catechol, an estrogen metabolite with no estrogenic activity. In parallel, in another mutually exclusive pathway 16α-hydroxylation produces metabolites with strong estrogenic properties (11). Polymorphisms in *CYP1A1* may affect the activity of the produced enzyme and contribute to the etiology of leiomyoma. A common variant in this gene is A>G in codon 462 (Ile462Val). Association between Ile462Val polymorphism and UL has been reported in the Chinese women (12). In homozygous state, the polymorphism has been thought to increase the activity of *CYP1A1* enzyme (13). The current case-control study was conducted to examine the hypothesis that *ESR1*-351(XbaI), *ESR1*-397(PvuII) and *CYP1A1* (Ile462Val) polymorphisms are associated with the development of uterine leiomyoma in female patients from Charmahal and Bakhtiari province of Iran.

In this case-control study, we investigated 276 women diagnosed with UL at the Department of Gynecology at Shahrekord Hajar Hospital from 2010 to 2011. UL was diagnosed by transvaginal sonographic examination and confirmed by histological test after myomectomy or hysterectomy. All the participants were at reproductive age. The mean age of the subjects was 44 ± 5.7 years, and the mean body mass index (BMI) was 27.5 ± 4.2 kg/m^2^. The healthy control group included 157 women without any evidence of leiomyoma according to transvaginal ultrasound exam. There were no differences between the case and control groups in terms of age, BMI, ethnicity and menarche. Cases with genital tract neoplasms, abnormal uterine bleeding, adenomyosis, pregnancy, *ESR1* dependent cancers, rheumatoid arthritis, alcohol intake, permanent or recent use of oral contraceptives and smoking habit were excluded from the study. Informed consent was obtained from all participants before their incorporation in the study. The Ethics Committees of the Shahrekord University of Medical Sciences approved all procedures.

Genomic DNA was extracted from blood samples using a standard phenol/chloroform extraction method. The quality of DNA was evaluated by spectrophotometry for all the samples. *ESR1* -351 XbaI A/G and -397 PvuII T/C polymorphisms were assayed by the method of polymerase chain reaction (PCR) followed by restriction fragment length polymorphism (RFLP). PCR amplification was performed using primers formerly reported (12, 14).

Table 1 summarizes the primer sequences, PCR conditions, restriction enzymes, digestion products and their lengths. PCR and digestion products were separated by vertical non-denaturing 8% polyacrylamide gel and visualized by silver staining. To confirm the genotyping results, a subset of PCR products were examined by DNA sequencing, and the results were 100% concordant.

**Table 1:** The primer sequences, PCR conditions and Restriction enzymes for *ESR1*-351 A/G and -397 T/C and *CYP1A1*gene polymorphisms


Polymorphism	Primers	Annealing temp (˚C/s)	Restriction enzyme	product sizes(bp)

***ESR1*-351 (14)**				
**A/G XbaI**	F:5´CTGCCACCCTATCTGTATCTTTTCCTATTCTCC -3´	60/40	XbaI	GG genotype: 1374bp
	R:5´-TCTTTCTCTGCCACCCTGGCGTCGATTATCTGA-3´			AA genotype: 982+ 392bp
				AG genotype: 1374+982+392bp
***ESR1*-397 (14)**	F:5´-CTGCCACCCTATCTGTATCTTTTCCTATTCTCC 60/40	60/40	PvuII	TT genotype: 1374bp
	R:5´-TCTTTCTCTGCCACCCTGGCGTCGATTATCTGA-3´			
**T /C PvuII**	F:5´-CTGCCACCCTATCTGTATCTTTTCCTATTCTCC			CC genotype:937+ 437bp
	R:5´-TCTTTCTCTGCCACCCTGGCGTCGATTATCTGA-3´			
***CYP1A1* (12) Ile462Val (A/G)**	F:5´-GATCTGAGTTCCTACCTGA-3´	60/60	HincII	TC genotype: 1374+937+437bp
	R:5´-AAGAGAAAGACCTCCCAGCGGTCAA-3´			AA genotype: 139bp
				AG genotype: 139+116+ 23bp


References are given in parentheses after each polymorphism. F and R indicate forward and reverse primers.

Genotypes and allelic frequencies for individual polymorphisms were compared between cases and controls using the X^2^-test. P<0.05 was considered statistically significant. Hardy- Weinberg equilibrium was tested with a goodness of fit X^2^-test with one degree of freedom to compare the observed genotype frequencies with the expected genotype frequencies. The associations between both alleles and genotypes and disease risk were calculated using odds ratios (OR) with a 95% confidence interval (CI).

The allele and genotype frequencies of *ESR1*- 351A/G in the case and control groups are given in table 2. The proportion of women homozygous for the A allele (AA), heterozygous (AG), and homozygous for the G allele (GG) were 32.7, 46.4 and 19.9%, respectively in the leiomyoma group, and 35.1, 49, and 15.9%, respectively in the control group. These frequencies are not significantly different between the two groups. The *ESR1*-397T/C allele and genotype frequencies of women with UL and controls are shown in table 3. The allele frequencies for T and C alleles were not significantly different between the case and control groups (p=0.386) (Table 2).

The *CYP1A1* (Ile462Val) polymorphism frequencies in the control and UL patient groups are given in table 3. The AG genotype frequencies were 12.7 and 8.3% in the patients and controls, respectively. The allele frequencies for A and G alleles were 0.94 and 0.06% in the leiomyoma and 0.96% and not significantly different between the UL and control groups (p=0.174, Table 2).

**Table 2:** TAllele frequencies of *ESR1*-351 A/G and *ESR1*-397 T/C gene polymorphisms in women with leiomyoma and normal controls


Polymorphism	Cases n=276 No (%)	Control n=157 No (%)	OR (95% CI)	P value

***ESR1* -351 A/G Genotype frequency**				
**AA**	93 (32.7)	55 (35.1)	1.00 (ref)	
**AG**	128 (46.4)	77 (49)	0.938 (0.635-1.52)	0.939
**GG**	55 (19.9)	25 (15.9)	1.31 (0.73-2.32)	0.372
***ESR1* -351 A/G Allele frequency**				
**A**	314 (56.9)	187 (59.6)	1.00 (ref)	
**G**	238 (43.1)	127(40.4)	1.112 (0.842-1.479)	0.444
***ESR1*-397 T/C Genotype frequency**				
**TT**	78 (28.3)	50 (31.8)	1.00 (ref)	
**TC**	133 (48.2)	74 (47.2)	1.152 (0.731-1.816)	0.542
**CC**	65 (23.5)	33 (21)	1.223 (0.729-2.187)	0.405
***ESR1*-397 T/C Allele frequency**				
**T**	289 (0.52)	174 (0.55)	1.00 (ref)	
**C**	263 (0.48)	140 (0.45)	1.13 (0.856-1.494)	0.386


**Table 3:** The genotype and allele frequencies of polymorphisms of *CYP1A1* Ile462Val gene in women with leiomyoma and normal controls


*CYP1A1* Genotypes	Cases n=276 No (%)	Control n=157 No (%)	OR (95% CI)	P value

**Genotype frequency**				
**AA**	241 (87. 3)	144 (91.7)	1.00 (ref)	
**AG**	35 (12.7)	13 (8.3)	1.609 (0.824-3.141)	0.106
**Allele frequency**				
**A**	517 (0.94)	301(0.96)	1.00 (ref)	
**G**	35 (0.06)	13 (0.04)	1.567 (0.816 -3.009)	0.174


OR; Odds ratio, 95% CI 95% confidence interval and ref; Reference.

Our results did not show significant difference in allelic and genotypic distribution of *CYP1A1* Ile462Val polymorphism between the two groups. So far, contradictory results have been reported as regard to the specified polymorphisms and the risk of estrogen related diseases. Results of the present study are in accordance with some of these studies. In a study by Rosa et al. (15) no association between *CYP17A1* and *CYP19* gene polymorphisms and UL were observed. Shin et al. (16) did not find any association between *CYP1A1* MspI gene polymorphism and breast cancer risk in Korean women either. However, our findings conflict with previously published findings in Chinese, Egyptian and Caucasian women reporting an association between *CYP1A1* polymorphism and UL (12, 14, 17, 18).

In the study on *ESR1* gene, no significant association were observed between either allele frequency or genotype distribution of *ESR1*- 351A>G and *ESR1*-397T>C polymorphisms and UL. Various studies have shown that *ESR1* (XbaI) and *ESR1* (PvuII) polymorphisms are associated with many estrogen-dependent diseases, such as early menarche (18), breast cancer (19), osteoporosis (20), prostate cancer (21), endometriosis and adenomyosis (22). Hsieh et al. (14) showed that there was an association between *ESR1* (XbaI) and *ESR1*(PvuII) and UL risk in the Chinese women. In contrast, Villanova et al. (23) failed to observe any significant association between *ESR1* polymorphisms and UL in Brazilian women. The underlying source of discrepancies in the results among different studies could partly be the differences in genetic background among the studied populations. It is also possible that estrogen receptor gene polymorphisms are differentially in linkage disequilibrium with an as yet unknown functional variant that influences leiomyoma susceptibility (14). Besides, Leiomyoma is a complicated phenotype and different pathways may lead to its pathogenesis. Thus far, many risk factors such as age, premature menstruation, obesity, nulliparity, lifestyle and ethnicity have been known for disease pathogensis (24). Ethnicity is said to play a significant role in genetic regulation of estrogen, ER action and association of polymorphism to particular disease (25). Therefore, it is not surprising to find that *ESR1* polymorphisms have no association in some ethnic groups.

Thus, the investigated polymorphisms seem to have no role in the genetic susceptibility of the tumors in the studied subjects. Furthermore, the subtypes of the UL were not determined in this study. Further studies are needed to identify the possible different etiology of the UL subtypes. Also, role of gene-environment interactions in the pathogenesis of UL should be investigated.
